# Diagnostic and prognostic EEG analysis of critically ill patients: A deep learning study

**DOI:** 10.1016/j.nicl.2022.103167

**Published:** 2022-08-27

**Authors:** Stefan Jonas, Michael Müller, Andrea O. Rossetti, Stephan Rüegg, Vincent Alvarez, Kaspar Schindler, Frédéric Zubler

**Affiliations:** a Sleep-Wake-Epilepsy-Center, Department of Neurology, Inselspital, Bern University Hospital, University of Bern, Bern, Switzerland; b Department of Neurology, Lausanne University Hospital and University of Lausanne, Lausanne, Switzerland; c Department of Neurology, University Hospital Basel, Basel, Switzerland; d Department of Neurology, Hôpital du Valais, Sion, Switzerland

**Keywords:** Acute consciousness impairment, Coma, Intensive care, Prognostication, Diagnostic, EEG, Machine learning, Deep learning

## Abstract

Visual interpretation of electroencephalography (EEG) is time consuming, may lack objectivity, and is restricted to features detectable by a human. Computer-based approaches, especially deep learning, could potentially overcome these limitations. However, most deep learning studies focus on a specific question or a single pathology. Here we explore the potential of deep learning for EEG-based diagnostic and prognostic assessment of patients with acute consciousness impairment (*ACI*) of various etiologies. EEGs from 358 adults from a randomized controlled trial (CERTA, NCT03129438) were retrospectively analyzed. A convolutional neural network was used to predict the clinical outcome (based either on survival or on best cerebral performance category) and to determine the etiology (four diagnostic categories). The largest probability output served as marker for the confidence of the network in its prediction (“certainty factor”); we also systematically compared the predictions with raw EEG data, and used a visualization algorithm (Grad-CAM) to highlight discriminative patterns. When all patients were considered, the area under the receiver operating characteristic curve (AUC) was 0.721 for predicting survival and 0.703 for predicting the outcome based on best CPC; for patients with certainty factor ≥ 60 % the AUCs increased to 0.776 and 0.755 respectively; and for certainty factor ≥ 75 % to 0.852 and 0.879. The accuracy for predicting the etiology was 54.5 %; the accuracy increased to 67.7 %, 70.3 % and 84.1 % for patients with certainty factor of 50 %, 60 % and 75 % respectively. Visual analysis showed that the network learnt EEG patterns typically recognized by human experts, and suggested new criteria. This work demonstrates for the first time the potential of deep learning-based EEG analysis in critically ill patients with various etiologies of *ACI*. Certainty factor and post-hoc correlation of input data with prediction help to better characterize the method and pave the route for future implementations in clinical routine.

## Highlights

• We use deep learning to analyze EEGs of 358 acute critically ill patients.
• DL is effective for clinical EEG interpretation in real-world conditions.
• Confidence of the network in its own prediction correlates with the accuracy.
• Features learnt for classification resemble patterns from visual EEG interpretation.
• This study is a further step toward use of AI for EEG in clinical setting.

## 1 Introduction

Acute consciousness impairment (*ACI*) represents a particular challenging condition for neurologists and intensive medicine specialists. Caregivers try to identify the underlying etiology in order to implement specific treatments (Edlow et al., 2014, Traub and Wijdicks, 2016); in case of delayed recoveries, there is need to estimate the chances that the patient will recover (Ramos et al., 2020, Sandroni et al., 2018). Both diagnostic and prognostic assessments are based on multimodal approaches (Edlow et al., 2014, Rossetti et al., 2016). Because EEG reflects the functioning of brain neurons - the activity of which is necessarily perturbed in case of impaired consciousness - it appears suitable for diagnostic and prognostic tool regardless of the underlying etiology (Sutter et al., 2015, Young, 2000). In practice, however, the diagnostic role of EEG in critically ill patients is essentially limited to detecting non-convulsive status epilepticus or delayed vasospasm (Claassen et al., 2013), whereas as prognostic tool it is mainly used in post hypoxic/ischemic encephalopathy (HIE) (Nolan et al., 2021). The limited use of EEG is partly due to the progresses of neuro-imaging, but also to the methods of interpretation. Currently, EEGs are still visually analyzed by trained neurophysiologists. Consequently, many centers have restricted access to EEG especially during nights and holidays (Gelisse et al., 2021). In addition, visual analysis is time-consuming, has limited intra- and inter-rate agreements (Arends et al., 2017, Benbadis et al., 2009, Jing et al., 2019), and is inherently restricted to features recognized by humans (Meyer et al., 2021, Tjepkema-Cloostermans et al., 2013).

Computer-based analysis has been proposed to circumvent these limitations (Zubler et al., 2016, Gemein et al., 2020). Deep learning (DL) appears particularly promising, since it offers the advantage to find out by itself which EEG features are relevant for classification (“feature extraction”). As such, DL is not limited to instructions provided by human experts, and is thus more likely to unravel new important EEG characteristics. DL has been successfully used for EEG analysis for brain computer interfaces or for specific neurologic or psychiatric conditions (for comprehensive reviews see (Craik et al., 2019, Roy et al., 2019)). In the context of critical care, EEG has been mainly used for prognostication in patients with hypoxic-ischemic encephalopathy after cardiac arrest (Jonas et al., 2019, Tjepkema-Cloostermans et al., 2019) or with epileptiform activity (Zafar et al., 2021). However, all these applications are somehow artificial in that only a specific question or a single pathology is considered at the time. While DL performs very well on these narrowly defined patient groups, real-word applications will require the ability to function on a broader set of (sometimes overlapping) pathologies and under noisier conditions, and without the need to redesign the network architecture before each application.

Here, we train a DL network on a prospectively acquired cohort of critically ill patients with *ACI* of various etiologies to predict both etiology and outcome. We also set out to quantify the confidence of the network in its predictions and to understand the reasons for the decision by comparing the predictions of the network with the raw EEG data, which represent crucial steps before a potential clinical use in the near future.

## 2 Material and methods

### 2.1 Data acquisition

Clinical and electroencephalographic data were acquired during the multicentric study CERTA (Continuous EEG Randomized Trial in Adults; NCT03129438). Details of the study have been published elsewhere (Rossetti et al., 2018, 2020). In summary, critically ill patients > 18 y with acute consciousness impairment (GCS ≤ 11 or FOUR ≤ 12) of any etiology hospitalized on the Intensive or Intermediate Care Units of four Swiss hospitals (Lausanne University Hospital/CHUV, Bern University Hospital/Inselspital, Basel University Hospital/USB, Sion Hospital) for whom an EEG was performed for medical reasons were included. As per design of the original study, patients who had electroencephalographic or clinical signs for epileptic seizures in the last 36 h or status epilepticus in the last 96 h were excluded, along with patients who were in a palliative situation. Immediately after inclusion, patients were randomized to undergo either a continuous EEG or two standard (20–30 min) EEGs. For the present study we only considered the first EEG (or a segment during the first 2 h in case of continuous EEG). The original study and the present post-hoc analysis were approved by the local ethic commissions (Project-ID 2017–00268).

Video-EEGs were recorded with a NicoletOne system (Viasys Neurocare, Madison WI, USA). Usually, 21 or 23 electrodes according to the international 10:20-system were used; however, in neurosurgical patients, several electrodes could be omitted due to bandages or drainages. For the present study we included the largest set of electrodes present in all patients, consisting of 9 electrodes placed at positions Fp1/2, T7/8, C3/4, O1/2, Cz (the reference electrode was placed near Fpz or Cz depending on the hospital). Five minutes in absence of external stimulation were selected. No EEG was excluded because of muscle, ECG, blinking or movement artifacts. The original sampling rate was usually 250 Hz (in some cases 1000 Hz). Prior to analysis the signals were down-sampled to 50 Hz and a high pass filter with cut-off frequency of 0.5 Hz was applied.

### 2.2 Defining prognosis and etiology

A detailed presentation of the prognostic and diagnostic categories can be found in (Müller et al., 2020). In short, we used two different binary prognostic categorizations, namely survival vs death at 6 months, and favorable vs unfavorable functional outcome based on the Cerebral Performance Category (CPC) assessed at 4 weeks and 6 months, whereby the best value was used (“best CPC”), and dichotomized into a favorable (CPC 1 or 2) or unfavorable outcome (CPC 3–5). For etiology, we used a categorization comprising four different diagnostic classes (based on the clinical information prospectively collected during the first week after inclusion): 1) *Stroke:* ischemic or hemorrhagic strokes, including non-traumatic subarachnoid hemorrhage; 2) *TBI:* traumatic brain injury and other non-vascular neurosurgical conditions; 3) *MIII:* metabolic disturbance, intoxication, infection, or inflammation; 4) *HIE*: Hypoxic-ischemic-encephalopathy after cardiac arrest. In case of multiple etiologies, the more “severe” condition was chosen (e.g. a patient with both HIE and metabolic disturbance was categorized as HIE; two patients with two equally severe conditions from different categories were excluded). Patients for whom the etiology was not known during the first week after inclusion were excluded from the diagnostic prediction.

### 2.3 Deep learning pipeline and network architecture

We used the *t-VGG GAP* architecture described in (Jonas et al., 2019). Hyper-parameters were not optimized for the present study. The 1D-CNN network consisted of three blocks, each containing two convolutional layers and one max-pooling layer, followed by a global average pooling layer and an output layer. The output layer consisted of a single neuron (with sigmoid activation function) when predicting the outcome; it consisted of four neurons (with softmax activation function) when predicting the etiology. The input was one-dimensional, corresponding to the voltage in *µ*V for a 10-second epoch, with nine channels (1 × 500 × 9). Each EEG was decomposed into 10-second epochs with 75 % overlap, which were presented independently to the network; the overall prediction for one EEG patient was obtained by averaging the prediction of all its epochs. We performed a stratified 5-fold cross-validation to ensure that no patient was classified by a network it helped training, using each time 80 % of the patients for training the network and the remaining 20 % as the test group. Each fold contained the same percentage of each class. For training, we used the Adam optimizer to minimize the cross-entropy loss function. The DL network was implemented in Python using the Keras framework (https://keras.io) v2.3.1; the code is provided as Supplementary Material.

### 2.4 Model output and certainty factor

When predicting the prognosis, the value of the final output neuron encoded the predicted probability *p* for survival or for favorable outcome. Accordingly, the probability for death or unfavorable outcome was *1-p*. The cutoff to make a binary prediction was set at *p = 0.5*. We introduced the *certainty factor,* defined as the maximum class probability *max(p, 1-p),* as a straightforward way to estimate the confidence of the network in its own prediction. When predicting the etiology, the output was a four-dimensional vector *(p_1_, p_2_, p_3_, p_4_)* encoding the probability distribution over all four diagnostic groups. Similarly, we defined the certainty factor as *max_i_(p_i_).* For both tasks we computed the detailed performance for all patients, as well as for the subgroups of patients classified with a certainty factor ≥ 0.6 and ≥ 0.75 (without retraining the network).

### 2.5 Explainability

Two different methods were used to explain a posteriori the decisions taken by the network. First, we analyzed visually all EEGs containing epochs classified (correctly or not) with a certainty factor ≥ 0.9. We restricted the analysis to the outcome classification based on best CPC. Second, we used the so-called Gradient-weighted Class Activation Mapping (Grad-CAM) algorithm (Selvaraju et al., 2017). This method quantifies how the classification result would be affected if different temporal segments within an epoch were to be modified, indicating which segments were discriminative for a specific class. For predicting the outcome, since the network had a single output neuron, each temporal segment was discriminative for a single class (favorable or unfavorable). For predicting the etiology, since the network had four output neurons, thus the same temporal segment could be discriminative for more than one class.

## 3 Results

### 3.1 Patients

364 patients were recruited, for details see (Rossetti et al., 2020). For 6 (1.7 %) the EEG could not be exported (missing or corrupted file), resulting in the inclusion of 358 patients for the present study (32 % female, median age 67 years (interquartile range 55–75), mean age 64 +/- 15). The etiology of *ACI* was known in 277 patients. Patients demographics are shown in Table 1.

**Table 1:** *Patients demographics.* For continuous values median and interquartile range are presented.

Etiology	N	Age [years]	Female (%)	Favorable Outcome (%)	Survival (%)	EEG delay since admission [h]
Stroke	80	67 [55 78]	38 (48)	21 (26)	38 (48)	69 [37 122]
TBI	47	63 [41 74]	12 (26)	21 (45)	30 (64)	75 [43 113]
MIII	44	64 [54 73]	13 (30)	20 (45)	25 (57)	140 [48 279]
HIE	106	66 [54 75]	28 (26)	34 (32)	42 (40)	24 [17 49]
No etiology available at recruitment time	81	68 [58 75]	26 (32)	40 (49)	50 (62)	102 [34 193]
All	358	67 [55 75]	117 (33)	136 (38)	185 (52)	60 [24 139]

### 3.2 Classification performance

**Fig. 1:**
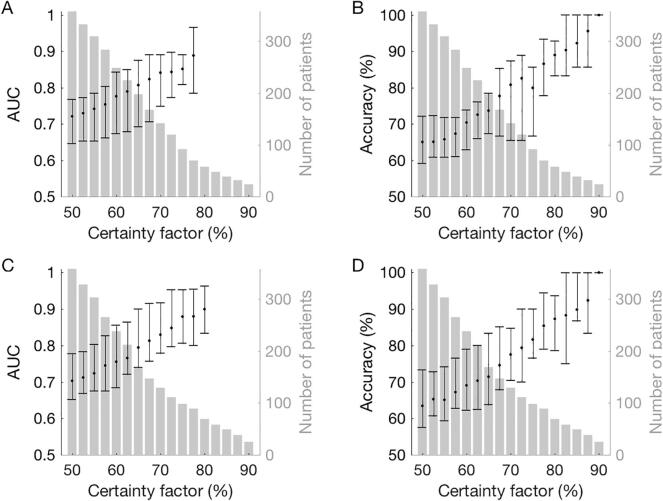
Performance and number of patients included for different values of certainty factor. (A) AUC (area under the ROC curve) for predicting survival. (B) Accuracy for predicting survival. (C) AUC for predicting favorable outcome. (D) Accuracy for predicting survival. Error bars indicate the range over the 5 cross-validation trials. Due to an overrepresentation of mortality and unfavorable outcome in EEGs classified with high certainty factor, the AUC could not be reliably computed for a certainty factor > 80 %.

*Outcome Prediction:* The detailed performance of the DL network for predicting the clinical outcome is presented in Table 2. When considering all patients (corresponding to a certainty factor of 50 %), the AUC was 0.721 for predicting survival and 0.703 for predicting a favorable outcome. Restricting analysis to patients for whom the certainty factor was at least 60 % increased the AUC to 0.776 for survival and 0.755 for favorable outcome; for patients with a certainty factor of at least 75 % the AUCs were 0.852 and 0.879, respectively. The performance for other values of certainty factors is illustrated in Fig. 1.

**Table 2:** *Performance of the deep-learning network for predicting the clinical outcome*, either survival vs death at 6 months, or favorable vs unfavorable outcome (based on best CPC). Average and range of five cross-validation trials. (PPV, positive predictive value; NPV, negative predictive value).

Subgroup	AUC	Accuracy	Sensitivity	Specificity	PPV	NPV
Predicting survival						
all (N = 358)	0.721	65.1	66.5	63.6	66.2	64.4
	[64.6 76.8]	[59.2 72.2]	[51.4 75.7]	[54.3 71.4]	[63.3 73.0]	[56.1 71.4]
certainty factor ≥ 60 % (N = 249)	0.776	70.4	72.8	67.8	68.5	72.7
	[67.5 84.3]	[63.0 74.0]	[65.2 84.0]	[59.3 73.1]	[62.1 74.1]	[64.0 80.0]
certainty factor ≥ 75 % (N = 92)	0.852 [81.0 89.1]	80.0 [66.7 85.7]	71.6 [57.1 87.5]	84.3 [70.6 90.9]	71.6 [44.4 88.8]	84.7 [80.0 90.9]
Predicting favorable outcome						
all (N = 358)	0.703	63.4	65.5	62.2	52	74.8
	[65.2 77.7]	[57.5 73.2]	[51.9 77.8]	[53.3 75.0]	[45.9 63.3]	[69.4 80.5]
certainty factor ≥ 60 % (N = 238)	0.755	69.0	70.8	67.6	55.0	81.4
	[68.4 85.5]	[62.2 79.1]	[46.7 85.0]	[59.4 82.8]	[43.8 66.7]	[72.4 87.6]
certainty factor ≥ 75 % (N = 98)	0.879 [80.5 95.2]	81.6 [76.5 90.0]	85.5 [66.7 100]	81.3 [73.3 85.7]	63.1 [33.3 75.0]	92.2 [83.3 100]

**Fig. 2:**
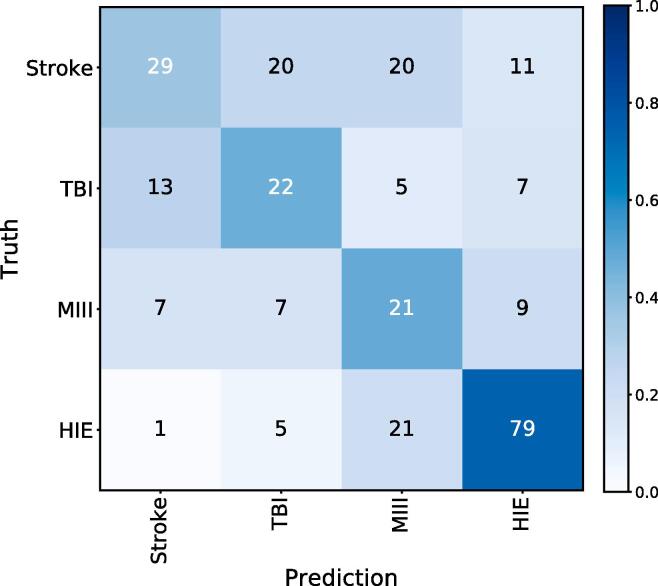
Confusion matrix for predicting the etiology. The heatmap values represent the relative prediction occurrence of a class when given a true label, i.e. the heatmap values sum to one across each row.

*Etiology:* The truth table for predicting the etiology is shown in Fig. 2. When all patients were considered (certainty factor of 25 %), the correct etiology was predicted in 54.4 % of patients; the accuracy was 36.3 % for patients with stoke, 46.8 % for TBI, 47.7 % for MIII, 74.5 % for HIE. Here also, considering only patients with a higher certainty factor increased the accuracy (Table 3).

**Table 3:** *Performance of the deep-learning network for predicting the etiology*. Average and range of five cross-validation trials.

Predicting etiology	
Subgroup	Accuracy
all (N = 277)	54.5
	[42.6 73.7]
certainty factor ≥ 50 % (N = 139)	67.6
	[53.8 76.0]
certainty factor ≥ 60 % (N = 91)	70.3 [61.5 75.0]
certainty factor ≥ 75 % (N = 44)	84.1
	[71.4 100.0]

### 3.3 Visual analysis of epochs classified with high confidence

**Fig. 3:**
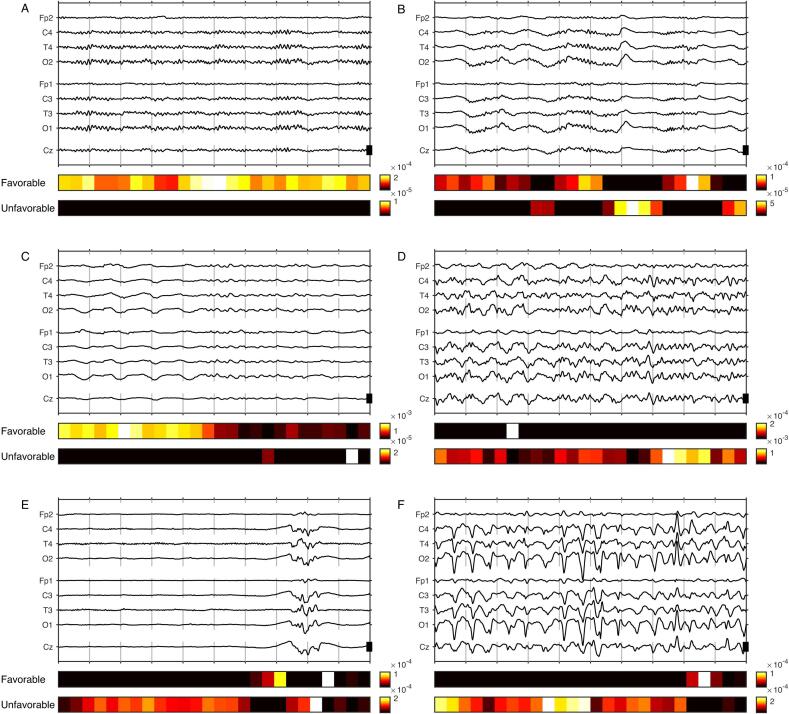
*EEG features discriminative for favorable or unfavorable outcome.* 10-second EEG epochs in reduced (9 electrodes) pseudo-monopolar montage; the scale bar represents 100 µ*V*. The two color bars under each EEG indicate the temporal segments recognized by the Grad-CAM algorithm as class-discriminative for favorable (upper bar) or unfavorable (lower bar) outcome [arbitrary units]. (A) Well-modulated alpha activity with posterior-anterior gradient discriminative for favorable outcome (M, 75y, hypoxic ischemic encephalopathy, CPC 2, predicted probability for favorable outcome (P) 0.98). (B) Rhythmic monomorphic fast activity (sleep spindles) discriminative for favorable outcome; in the same epoch a K-complex was slightly discriminative for unfavorable outcome (M, 47, hypoxic ischemic encephalopathy, CPC 1, P 0.98). (C) As illustrated in this example, generalized rhythmic delta activity (G-RDA) was much more suggestive of favorable outcome than rhythmic theta activity (M, 77y, delayed awakening after surgery not involving the brain, CPC 2, P 0.90). (D) Diffuse monomorphic theta activity of relatively high amplitude, without clear modulation or posterior-anterior gradient, discriminative for unfavorable outcome (F, 56y, ischemic stroke, CPC 5, P 0.07). (e) Suppressed segment and epileptiform bursts discriminative for unfavorable outcome; in this particular example the slower and “smooth” parts at the beginning and end of the burst were discriminative for favorable outcome. (M, 88y, hypoxic ischemic encephalopathy, CPC 5; P 0.05). (f) Triphasic waves were often predictive for unfavorable outcome, especially when sharply contoured as in this example (F, 55y, subarachnoid hemorrhage, CPC 1, P 0.03 [i.e. false negative]).

157 EEGs contained at least one epoch classified with a certainty factor of ≥ 0.9, of which 68 were *true positives* (TP) for favorable outcome. In those TP epochs, the background was continuous with a single predominant frequency in 40 cases (e.g. Fig. 3A), continuous with mixed or alternating frequencies in 20 cases (often with short segments of monomorphic rhythmic activity, e.g. Fig. 3BC), discontinuous in 7 cases. In one case the background could not be characterized because of opened eyes. The only rhythmic or periodic patterns (Hirsch et al., 2021) were generalized rhythmic delta activity (G-RDA, present in 18 cases; Fig. 3C) and generalized periodic triphasic discharges (2 cases). Eye blinking artifacts were present in 8 cases.

Nineteen EEGs contained *false positives (FP)* with a certainty factor ≥ 0.9*.* The patterns were very similar to those in TP: continuous monomorphic rhythmic activity in 10 cases, continuous mixed frequency activity in 8 cases; one EEG contained repetitive epileptic seizures, of which the epochs at the beginning of the seizures displaying low amplitude fast activity were falsely classified as favorable outcome. As for TP, several FP epochs contained G-RDA and triphasic discharges.

Eighty-three EEGs contained *true negatives (TN)*: 20 cases had a suppressed background; 9 an attenuated background; 25 a burst-suppression (Fig. 3E); 23 a continuous irregular background (often with fast on slow activity, whereby the fast activity was of higher amplitude than for TP or FP; Fig, 1D), 5 cases were seizures or status epilepticus, and one EEG was continuous with sporadic interictal discharges. Rhythmic or periodic patterns found in TN epochs were lateralized rhythmic delta activity (1 case) and triphasic periodic discharges (2 cases).

Finally, 34 EEGs contained *false negatives* (FN), which appeared similar to those in TN: 2 cases were attenuated background, 5 were burst-suppression, 29 irregular continuous background, 3 seizures, 4 sharply contoured triphasic periodic discharges (Fig. 3F), 4 cases contained large movement artifacts, 3 a high amplitude EMG artifact, and 3 showed a interhemispheric asymmetry in frequency and/or amplitude.

### 3.4 Discriminative features for predicting outcome

GradCAM provided two heat maps for favorable and unfavorable outcome (Fig. 3), which confirmed that the EEG patterns observed in epochs classified with high certainty were indeed relevant for prediction. In particular, posterior dominant rhythm (Fig. 3A), short segments of monomorph low amplitude fast activity such as sleep spindles (Fig. 3B), or generalized rhythmic delta activity (Fig. 3C), were discriminative for favorable outcome. By contrast, polymorphic (especially theta) activity, mixed frequencies with temporal and spatial heterogeneity (including focal slowing) or faster activity of higher amplitude superimposed on slow waves were usually discriminative for an unfavorable outcome (Fig. 3D). Suppressed segments and epileptiform activity were discriminative for unfavorable outcome (Fig. 3E). Triphasic waves were associated with unfavorable outcome when fast and sharply contoured (Fig. 3F), but could be discriminative for favorable outcome when broader and of lower amplitude. Eye blinking artifacts were discriminative for favorable, muscle artifact usually for unfavorable outcome. In many segments, however, it was not possible by inspecting the EEG data to identify the reasons for the Grad-CAM result.

### 3.5 Discriminative features for predicting etiology

**Fig. 4:**
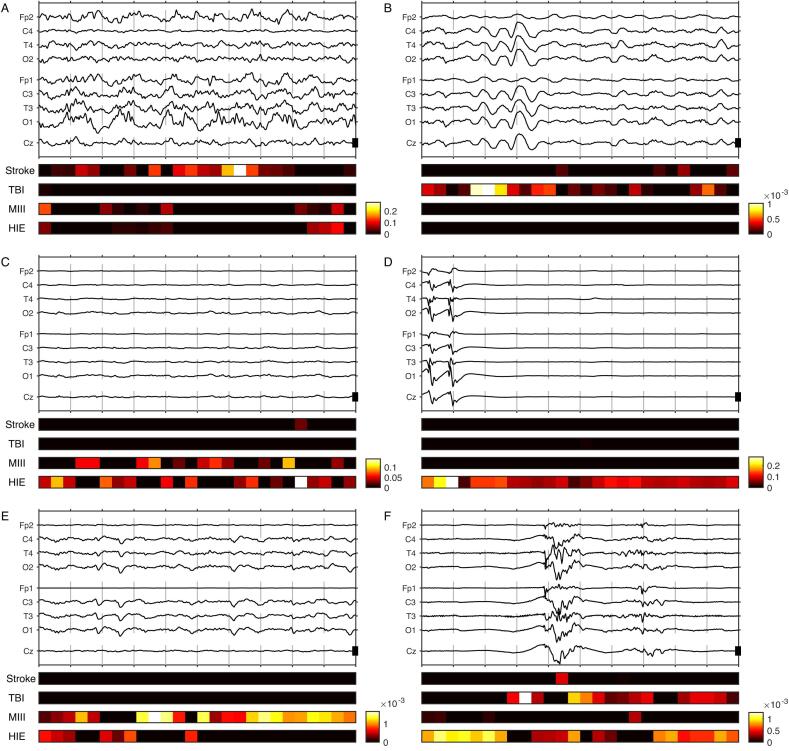
*EEG features discriminative for specific etiologies*. 10-second EEG epochs in reduced (9 electrodes) pseudo-monopolar montage; the scale bar represents 100 µ*V*. Color bars indicate the temporal segments discriminative for the four etiology groups: Stroke, traumatic brain injury or other neurosurgical diagnosis (TBI); metabolic, intoxication, infectious or inflammatory disorders (MIII); hypoxic-ischemic encephalopathy (HIE) [arbitrary units]. (A) Focal slowing posterior left discriminative for stroke (F, 66y, probability for stroke 0.96). (B) High amplitude rhythmic delta waves discriminative for TBI (M, 21y, TBI, probability for TBI 0.90) (C) Mixed frequencies of reduced amplitude discriminative for MIII or HIE (M, 75, metabolic encephalopathy, probability for MIII 0.76) (D) Suppressed segments and highly epileptiform activity strongly discriminative for HIE (M, 65, HIE, probability for HIE 83 %). (E) Continuous variable background with a few triphasic configured transients; the temporal segments were discriminative for MIII and/or HIE (80, F, true diagnostic HIE, probability for MIII 0.76 [i.e. error]). (F) This EEG epoch contains features that typically discriminate all four etiologies: The temporal segment with largest left–right asymmetry (between channels T3 and T4 in middle of the burst) was discriminative for stroke; the slow delta component of the same burst was discriminative for TBI; the polymorph, non-epileptiform theta activity in the second burst was suggestive of MIII; suppressed and polymorph epileptiform segments were strongly discriminative for HIE (M, 65, true diagnostic HIE, probability for stroke 0.37, probability for HIE 0.36 [i.e. error]).

Grad-CAM provided four maps, one for each diagnostic class (Fig. 4). A left–right asymmetry was usually discriminative for stroke (Fig. 4A); high amplitude delta activity was often associated to TBI (Fig. 4B); a mixed frequency background was usually discriminative either for MIII (when of high amplitude, or with triphasic transients) or for HIE (Fig. 4CD). Segments with a suppressed background or highly epileptiform activity were systematically discriminative for HIE (Fig. 4DF). Often, these different elements were found within the same epoch. Here also, in many cases it was difficult to understand the reasons for the GradCAM results.

## 4 Discussion

In this work we trained a deep-learning network to predict the clinical outcome of patients with *ACI* of various etiologies based on the EEG recorded on the intensive or intermediate care unit. The network architecture was not optimized for the present study, but taken unmodified from a previous study on prognostication in patients with HIE (Jonas et al., 2019). When considering all patients, the AUCs were slightly above 0.7 for both outcome measures; when restricting the analysis to patients with a certainty factor ≥ 60 %, the AUCs increased to ca. 0.75, and for a certainty factor ≥ 75 % to over 0.85.

Most studies using computational methods for EEG-based prognostication in the ICU are conducted on homogeneous groups, typically on patients with HIE (Beudel et al., 2014, Tzovara et al., 2016, Zubler et al., 2017, Tjepkema-Cloostermans et al., 2017, Tjepkema-Cloostermans et al., 2019, Jonas et al., 2019, Alnes et al., 2021). Only a handful have been conducted on patients with impaired consciousness of various etiologies, using predefined visual (Müller et al., 2020, Zhang et al., 2011, Vassallo et al., 2021, Kang et al., 2015, Selioutski et al., 2019) or quantitative (You et al., 2018, Zubler et al., 2016) or mixed (Zafar et al., 2021) features. To the best of our knowledge, it is the first time that DL is used for this task.

In a previous work on the same cohort, a mixed approach was taken whereby a random forest classifier (RF) was used to predict the clinical outcome based on pre-defined EEG features scored visually by human experts (Müller et al., 2020). The mixed visual/RF approach reached a higher AUC (0.812 for survival, 0.79 for favorable outcome) than the DL network when applied to all patients (i.e. irrespective of the certainty factor). Several reasons may explain this discrepancy: First, in the visual-RF approach, classification was based on the presence of standardized visual EEG-features (Hirsch et al., 2013) scored by neurophysiologists with specific training for intensive care EEG. Thus, the algorithm relied heavily on pre-existing theoretical and practical knowledge gathered by experts over many years. By contrast, the DL network learned within few minutes based on 246 EEGs (the amount of training data in each of the 5 cross-validation trials) without any previous knowledge or explicit instruction from the programmers. Second, background reactivity was one of the features provided to the RF classifier (it became the single most important feature for outcome prediction when patients of all etiologies were included). The DL network did not benefit from this important clue since only EEG recordings without stimulus were used for this study. Finally, RF is known to be particularly efficient with small data sets, because the risk of overfitting is low. By contrast, since it performs both feature extraction and classification, DL is particularly prone to overfitting and typically requires larger amount of data (Aellen et al., 2021). Conducting a similar analysis using thousands of patients may improve a dl-based algorithm performance (Jing et al., 2019, van Leeuwen et al., 2019). The extreme variability in EEG patterns found in patients with various pathologies probably further increases the risk of overfitting. Indeed, the same DL architecture performed extremely well when applied to a more homogeneous population of patients with HIE (AUC of 0.90) (Jonas et al., 2019).

The versatility of DL is illustrated by the fact that the same network architecture, with four neurons instead of one in the output layer, could be trained to predict the etiology of *ACI*. When all patients were included, the accuracy was of 54 % (at a chance level of 25 %). Even though the accuracy increased for subgroups of patients with higher certainty factor, the performance might seem limited. However, the task was complicated by the overlap in diagnostic categories (for instance, a patient with HIE could also have a metabolic disturbance or an infection, but only the first category would be considered as correct). More importantly, diagnostics based on the sole EEG is a very difficult task. In current clinical practice the diagnostic role of EEG is often restricted to seizure detection (Herman et al., 2015). A few studies used visual (for instance based on the Young scale (Alkhamis and Nazish, 2020)) or quantitative (using signal correlation measures (Zubler et al., 2016)) analysis for comparing patients with various etiologies at group level, however no attempt was made for classification of individual subjects.

The accuracy was better for HIE than for the other diagnostic categories. This is not surprising considering that in this pathology the cerebral grey matter is primarily lesioned, that is, the very structure producing the EEG signal. Furthermore, some EEG patterns (such as a suppressed background) were only found in this category. Finally, it is the category with the largest number of patients, so that learning might be more robust.

Artificial intelligence, and DL in particular, has often been used as a “black box”. For medical applications, however, trust and explainability are of paramount importance (Kundu, 2021, Markus et al., 2021). We have addressed these crucial points by introducing the certainty factor and by confronting the network decisions with visual analysis.

The certainty factor, corresponding to the prediction probability attributed to the winning class, was used to quantify the network's trust in its own prediction. More complex alternatives exist (Gal and Ghahramani, 2016) and probabilities expressed by modern DL network do not usually correspond exactly to real probabilities (Guo et al., 2017). Despite its theoretical limitations the certainty factor was clearly related to the performance: When all patients were considered (corresponding to a certainty of 50 %), prognostication was accurate in about 2/3 of cases. By contrast, in the subgroup of patients with a certainty factor ≥ 75 %, 4/5 predictions were correct - but the number of predictions that could be made was reduced. The same applied for diagnostics. This type of reasoning is familiar to clinicians, who are used to distinguish the situations where the result of a test is useful, from those where the results lie in a grey area and do not contribute to the clinical assesment.

The certainty factor, however, was provided by the network itself, and could thus not be used to exclude biases in the training process. Moreover, it did not explain on which criteria each particular subject has been classified (“explainability”). Therefore, we compared the network output with the EEG given as input. Visual examination of epochs classified with high confidence confirmed the presence of features used also by human specialists, such as a continuous rhythmic background (Beuchat et al., 2021, Hofmeijer et al., 2015, Westhall et al., 2016), sleep spindles (Vassallo et al., 2021), G-RDA (Beuchat et al., 2021) in epochs classified as favorable outcome; or such as suppressed background and burst-suppression (Hofmeijer et al., 2015, Ruijter et al., 2019, Westhall et al., 2016), or epileptiform activity (Zafar et al., 2021) in epochs classified as unfavorable outcome.

The presence of these typical features in misclassified EEGs can explain some of the errors: A suppressed EEG in a patient with favorable outcome could be due to sedation, whereas a continuous rhythmic background with posterior to anterior gradient would also be recognized by a human expert as heralding favorable outcome, even though the patient might suffer a later complication.

Also, eye blinking and movement artifacts were found in epochs classified as favorable outcome, which makes sense, since a moving patient usually indicates a less deep coma. Incidentally, this is a reminder that biological artifacts can contribute to the clinical assessment (Caporro et al., 2019).

The use of Grad-CAM allowed us to refine our explainability analysis by ensuring that not only was a typical pattern present in an epoch, but that the time frame when it occurred was important for decision-making. Grad-CAM also suggested at least one new diagnostic criteria that will have to be confirmed in future studies, namely the relative amplitude of superimposed fast on low activity - low amplitude being discriminative for favorable (Fig. 3B) and larger amplitude for unfavorable outcome (Fig. 3DE). We and others have already shown that Grad-CAM can be used with EEG (Jonas et al., 2019, Li et al., 2020). The present study illustrates that Grad-CAM also works for prognostication when multiple etiologies are involved, as well as for diagnostic predictions with non-binary classes. It is important to mention, however, that saliency maps methods such as Grad-CAM highlight regions with discriminative features, but do not explain why these features are discriminative (Ghassemi et al., 2021), so that currently interpretation by a expert is still needed.

DL has become the standard technology for automatic translation, fraud detection, self-driving cars. Whether it will play an important role in clinical decision-making in the future remains an open a question. Besides obvious considerations on cybersecurity and quality of training data, the technology would have to become more accessible to medical doctors without a computer science background, which means a friendly user interface, allowing continuous learning tailored to local conditions, but without the need to reprogram the algorithm for each specific situation. This was our motivation for taking an existing architecture and train it using new data without re-optimizing the hyper parameters. Explainability will be necessary to inform patients and family and to ensure that continuous training is not done on artifacts. Finally, since most medical decisions (especially decision to withdraw life supporting treatment in comatose patients) are taken using multiple clinical and paraclinical modalities, one has to accept that a dl-system using a single modality will not reach the extremely high performance seen in other dl-applications. However, this does not mean that the information provided by a dl-based EEG reader could not be useful in a clinical setting, once its sensitivity and specificity have been well characterized. For instance the pupillary light reflex has a poor sensitivity for predicting unfavorable outcome, but can still be very useful when combined with other tests (Oddo et al., 2018). A further step would be to integrate these different modalities (Moeskops et al., 2016, van Leeuwen et al., 2019).

Our study has several limitations, most of which derive from the design of the original prospective study during which the data was acquired. First, patients with recent status epilepticus or seizures prior to enrolment were excluded. Accordingly, we did not incorporate a category defined by an epileptic origin of *ACI*. Other studies have shown that DL can be effective in detecting epileptiform activity (Craik et al., 2019, Roy et al., 2019). The cohort size is relatively large for prognostication studies using intensive care EEGs, but is still relatively low for typical DL studies. Self-fulfilling prophecy is a risk in all prognostication studies. However, we estimate that this risk was low for the present study since we analyzed the first EEG, whereas only the second EEG could be used along with several other criteria (listed in (Caporro et al., 2019)) for decisions to withdraw life-supporting therapy (WLST) in patients with HIE. Moreover, EEG was not used to determine WLST in other etiologies. Finally, the definition of diagnostic classes was performed a posteriori, and contained very broad categories with clear overlap, such as metabolic disturbance or infection in patients with other categories.

The strengths of our study are that the data were carefully acquired prospectively. Since the decision to perform an EEG was made by treating physicians based on clinical considerations only, the population is very likely to resemble real-world situations where an EEG interpretation is required (Rossetti et al., 2020). Importantly, we did not exclude patients because of the presence of artifacts. Finally, we did not consider performance only, but took into account the degree of certainty with which a prediction was made, and systematically compared the input and the prediction to gain insight in the criteria learnt by the network - two aspects which are often neglected in DL studies.

## 5 Conclusion

In this work we applied deep learning to a real-world complex clinical problem. We found that after training on a few hundred samples, a basic convolutional neural network was capable of interpreting EEGs from patients with acute consciousness impairment of various etiologies. We showed that the maximum output probability is a simple and efficient way to determine the confidence of the network in its own prediction, and that this confidence reflects the accuracy. Finally, we confirmed that a saliency map algorithm such as GradCAM was effective for EEG, even for non-binary categories. This visualization algorithm allows the neurologists and electrophysiologists to verify that the network uses legitimate EEG features (and not, for instance, non-biological artifact). It also helps explaining errors and limitations of the methods, and - possibly - discovering new EEG patterns. The present study represents a further step towards future inclusion of DL in clinical settings.

## Funding

The Swiss National Science Foundation supported this study (grant 320030_169379 to AOR, SR, VA, KS); the funding agency had no role in design of the study, analysis or decision to publish.

## 7 Data and material availability

Free release of patient information and EEG recordings has not been granted for further research by the local Ethics Commissions. Data can be obtained for verification purposes from the corresponding author.

## 8 Code availability

Code for the network and GradCAM implementation are provided as Supplementary Material.

## CRediT authorship contribution statement

**Stefan Jonas:** Conceptualization, Methodology, Software, Writing – original draft. **Michael Müller:** Data curation, Writing – review & editing. **Andrea O. Rossetti:** Resources, Data curation, Writing – original draft. **Stephan Rüegg:** Resources, Writing – review & editing. **Vincent Alvarez:** Resources, Writing – review & editing. **Kaspar Schindler:** Resources, Writing – original draft. **Frédéric Zubler:** Conceptualization, Methodology, Resources, Data curation, Writing – original draft, Supervision.

## Declaration of Competing Interest

The authors declare that they have no known competing financial interests or personal relationships that could have appeared to influence the work reported in this paper.

## References

[b0005] Aellen F.M., Göktepe-Kavis P., Apostolopoulos S., Tzovara A. (2021). Convolutional neural networks for decoding electroencephalography responses and visualizing trial by trial changes in discriminant features. J. Neurosci. Methods.

[b0010] Alkhamis F., Nazish S. (2020). Electroencephalographic grading of neuronal dysfunction in various etiologies of encephalopathy. Clin. EEG Neurosci..

[b0015] Alnes S.L., Lucia M.D., Rossetti A.O., Tzovara A. (2021). Complementary roles of neural synchrony and complexity for indexing consciousness and chances of surviving in acute coma. NeuroImage.

[b0020] Arends J.B., van der Linden I., Ebus S.C., Debeij M.H., Gunning B.W., Zwarts M.J. (2017). Value of re-interpretation of controversial EEGs in a tertiary epilepsy clinic. Clin. Neurophysiol..

[b0025] Benbadis S.R., LaFrance W.C., Papandonatos G.D., Korabathina K., Lin K., Kraemer H.C., Treatment Workshop N.E.S. (2009). Interrater reliability of EEG-video monitoring. Neurology.

[b0030] Beuchat I., Rossetti A.O., Novy J., Schindler K., Ruüegg S., Alvarez V. (2021). Continuous versus routine standardized electroencephalogram for outcome prediction in critically Ill adults: analysis from a randomized trial. Crit. Care Med. Publish Ahead of Print..

[b0035] Beudel M., Tjepkema-Cloostermans M.C., Boersma J.H., van Putten M.J.A.M. (2014). Small-World Characteristics of EEG patterns in post-anoxic encephalopathy. Front. Neurol..

[b0040] Caporro M., Rossetti A.O., Seiler A., Kustermann T., Nguepnjo Nguissi N.A., Pfeiffer C., Zimmermann R., Haenggi M., Oddo M., De Lucia M., Zubler F. (2019). Electromyographic reactivity measured with scalp-EEG contributes to prognostication after cardiac arrest. Resuscitation.

[b0045] Claassen, J., Taccone, F.S., Horn, P., Holtkamp, M., Stocchetti, N., Oddo, M., Neurointensive Care Section of the European Society of Intensive Care Medicine, 2013. Recommendations on the use of EEG monitoring in critically ill patients: consensus statement from the neurointensive care section of the ESICM. Intensive Care Med. 39, 1337–1351. https://doi.org/10.1007/s00134-013-2938-4. 10.1007/s00134-013-2938-4 23653183

[b0050] Craik A., He Y., Contreras-Vidal J.L. (2019). Deep learning for electroencephalogram (EEG) classification tasks: a review. J. Neural Eng..

[b0055] Edlow J.A., Rabinstein A., Traub S.J., Wijdicks E.F.M. (2014). Diagnosis of reversible causes of coma. Lancet Lond. Engl..

[b0060] Gal, Y., Ghahramani, Z., 2016. Dropout as a Bayesian Approximation: Representing Model Uncertainty in Deep Learning, in: Proceedings of the 33rd International Conference on International Conference on Machine Learning - Volume 48, ICML’16. JMLR.org, pp. 1050–1059.

[b0065] Gelisse P., Genton P., Crespel A., Lefevre P.H. (2021). Will MRI replace the EEG for the diagnosis of nonconvulsive status epilepticus, especially focal?. Rev. Neurol. (Paris).

[b0070] Gemein L.A.W., Schirrmeister R.T., Chrabąszcz P., Wilson D., Boedecker J., Schulze-Bonhage A., Hutter F., Ball T. (2020). Machine-learning-based diagnostics of EEG pathology. NeuroImage.

[b0075] Ghassemi M., Oakden-Rayner L., Beam A.L. (2021). The false hope of current approaches to explainable artificial intelligence in health care. Lancet Digit. Health.

[b0080] Guo C., Pleiss G., Sun Y., Weinberger K.Q., Precup D., Teh Y.W. (2017). Proceedings of the 34th International Conference on Machine Learning. PMLR, Proceedings of Machine Learning Research.

[b0085] Herman, S.T., Abend, N.S., Bleck, T.P., Chapman, K.E., Drislane, F.W., Emerson, R.G., Gerard, E.E., Hahn, C.D., Husain, A.M., Kaplan, P.W., LaRoche, S.M., Nuwer, M.R., Quigg, M., Riviello, J.J., Schmitt, S.E., Simmons, L.A., Tsuchida, T.N., Hirsch, L.J., Critical Care Continuous EEG Task Force of the American Clinical Neurophysiology Society, 2015. Consensus statement on continuous EEG in critically ill adults and children, part I: indications. J. Clin. Neurophysiol. Off. Publ. Am. Electroencephalogr. Soc. 32, 87–95. https://doi.org/10.1097/WNP.0000000000000166. 10.1097/WNP.0000000000000166 PMC4435533 25626778

[b0090] Hirsch L.J., LaRoche S.M., Gaspard N., Gerard E., Svoronos A., Herman S.T., Mani R., Arif H., Jette N., Minazad Y., Kerrigan J.F., Vespa P., Hantus S., Claassen J., Young G.B., So E., Kaplan P.W., Nuwer M.R., Fountain N.B., Drislane F.W. (2013). American clinical neurophysiology society’s standardized critical care EEG Terminology: 2012 version. J. Clin. Neurophysiol..

[b0095] Hirsch L.J., Fong M.W.K., Leitinger M., LaRoche S.M., Beniczky S., Abend N.S., Lee J.W., Wusthoff C.J., Hahn C.D., Westover M.B., Gerard E.E., Herman S.T., Haider H.A., Osman G., Rodriguez-Ruiz A., Maciel C.B., Gilmore E.J., Fernandez A., Rosenthal E.S., Claassen J., Husain A.M., Yoo J.Y., So E.L., Kaplan P.W., Nuwer M.R., van Putten M., Sutter R., Drislane F.W., Trinka E., Gaspard N. (2021). American clinical neurophysiology society’s standardized critical care EEG Terminology: 2021 Version. J. Clin. Neurophysiol. Off. Publ. Am. Electroencephalogr. Soc..

[b0100] Hofmeijer J., Beernink T.M.J., Bosch F.H., Beishuizen A., Tjepkema-Cloostermans M.C., van Putten M.J.A.M. (2015). Early EEG contributes to multimodal outcome prediction of postanoxic coma. Neurology.

[b0105] Jing J., Sun H., Kim J.A., Herlopian A., Karakis I., Ng M., Halford J.J., Maus D., Chan F., Dolatshahi M., Muniz C., Chu C., Sacca V., Pathmanathan J., Ge W., Dauwels J., Lam A., Cole A.J., Cash S.S., Westover M.B. (2019). Development of expert-level automated detection of epileptiform discharges during electroencephalogram interpretation. JAMA Neurol.

[b0110] Jonas S., Rossetti A.O., Oddo M., Jenni S., Favaro P., Zubler F. (2019). EEG-based outcome prediction after cardiac arrest with convolutional neural networks: Performance and visualization of discriminative features. Hum. Brain Mapp..

[b0115] Kang X.-G., Yang F., Li W., Ma C., Li L., Jiang W. (2015). Predictive value of EEG-awakening for behavioral awakening from coma. Ann. Intensive Care.

[b0120] Kundu S. (2021). AI in medicine must be explainable. Nat. Med..

[b0125] Li Y., Yang H., Li J., Chen D., Du M. (2020). EEG-based intention recognition with deep recurrent-convolution neural network: Performance and channel selection by Grad-CAM. Neurocomputing.

[b0130] Markus A.F., Kors J.A., Rijnbeek P.R. (2021). The role of explainability in creating trustworthy artificial intelligence for health care: A comprehensive survey of the terminology, design choices, and evaluation strategies. J. Biomed. Inform..

[b0135] Meyer G.M., Marco-Pallarés J., Boulinguez P., Sescousse G. (2021). Electrophysiological underpinnings of reward processing: Are we exploiting the full potential of EEG?. NeuroImage.

[b0140] Moeskops P., Wolterink J.M., van der Velden B.H.M., Gilhuijs K.G.A., Leiner T., Viergever M.A., Išgum I., Ourselin S., Joskowicz L., Sabuncu M.R., Unal G., Wells W. (2016). Medical Image Computing and Computer-Assisted Intervention –.

[b0145] Müller M., Rossetti A.O., Zimmermann R., Alvarez V., Rüegg S., Haenggi M., Z’Graggen W.J., Schindler K., Zubler F. (2020). Standardized visual EEG features predict outcome in patients with acute consciousness impairment of various etiologies. Crit. Care Lond. Engl..

[b0150] Nolan J.P., Sandroni C., Böttiger B.W., Cariou A., Cronberg T., Friberg H., Genbrugge C., Haywood K., Lilja G., Moulaert V.R.M., Nikolaou N., Olasveengen T.M., Skrifvars M.B., Taccone F., Soar J. (2021). European resuscitation council and european society of intensive care medicine guidelines 2021: post-resuscitation care. Intensive Care Med..

[b0155] Oddo M., Sandroni C., Citerio G., Miroz J.-P., Horn J., Rundgren M., Cariou A., Payen J.-F., Storm C., Stammet P., Taccone F.S. (2018). Quantitative versus standard pupillary light reflex for early prognostication in comatose cardiac arrest patients: an international prospective multicenter double-blinded study. Intensive Care Med..

[b0160] Ramos, J.G.R., Dias, R.D., Passos, R. da H., Batista, P.B.P., Forte, D.N., 2020. Prognostication in urgent intensive care unit referrals: a cohort study. BMJ Support. Palliat. Care 10, 118–121. https://doi.org/10.1136/bmjspcare-2018-001567. 10.1136/bmjspcare-2018-001567 30171040

[b0165] Rossetti A.O., Rabinstein A.A., Oddo M. (2016). Neurological prognostication of outcome in patients in coma after cardiac arrest. Lancet Neurol..

[b0170] Rossetti A.O., Schindler K., Alvarez V., Sutter R., Novy J., Oddo M., Warpelin-Decrausaz L., Rüegg S. (2018). Does Continuous video-EEG in patients with altered consciousness improve patient outcome? current evidence and randomized controlled trial design. J. Clin. Neurophysiol. Off. Publ. Am. Electroencephalogr. Soc..

[b0175] Rossetti A.O., Sutter R., Rueegg S., Zubler F., Novy J., Warpelin-Decrausaz L., Alvarez V. (2020). Continuous versus routine EEG in critically ill adults with altered consciousness and no recent seizure: a multicenter randomized trial. JAMA Neurol..

[b0180] Roy Y., Banville H., Albuquerque I., Gramfort A., Falk T.H., Faubert J. (2019). Deep learning-based electroencephalography analysis: a systematic review. J. Neural Eng..

[b0185] Ruijter B.J., Tjepkema-Cloostermans M.C., Tromp S.C., van den Bergh W.M., Foudraine N.A., Kornips F.H.M., Drost G., Scholten E., Bosch F.H., Beishuizen A., van Putten M.J.A.M., Hofmeijer J. (2019). Early electroencephalography for outcome prediction of postanoxic coma: A prospective cohort study. Ann. Neurol..

[b0190] Sandroni C., D’Arrigo S., Nolan J.P. (2018). Prognostication after cardiac arrest. Crit. Care Lond. Engl..

[b0195] Selioutski O., Roberts D., Hamilton R., Ghosh H., Nickels J., Konig Toro F., Kruppenbacher M., Auinger P., Kaplan P.W., Birbeck G.L. (2019). Continuous EEG Monitoring predicts a clinically meaningful recovery among adult inpatients. J. Clin. Neurophysiol. Off. Publ. Am. Electroencephalogr. Soc..

[b0200] Selvaraju R.R., Cogswell M., Das A., Vedantam R., Parikh D., Batra D. (2017). In: 2017 IEEE International Conference on Computer Vision (ICCV). Presented at the 2017 IEEE International Conference on Computer Vision (ICCV).

[b0205] Sutter R., Kaplan P.W., Valença M., De Marchis G.M. (2015). EEG for Diagnosis and prognosis of acute nonhypoxic encephalopathy: history and current evidence. J. Clin. Neurophysiol. Off. Publ. Am. Electroencephalogr. Soc..

[b0210] Tjepkema-Cloostermans M.C., van Meulen F.B., Meinsma G., van Putten M.J. (2013). A Cerebral Recovery Index (CRI) for early prognosis in patients after cardiac arrest. Crit. Care.

[b0215] Tjepkema-Cloostermans M.C., Hofmeijer J., Beishuizen A., Hom H.W., Blans M.J., Bosch F.H., van Putten M.J.A.M. (2017). Cerebral recovery index: reliable help for prediction of neurologic outcome after cardiac arrest. Crit. Care Med..

[b0220] Tjepkema-Cloostermans M.C., da Silva Lourenço C., Ruijter B.J., Tromp S.C., Drost G., Kornips F.H.M., Beishuizen A., Bosch F.H., Hofmeijer J., van Putten M.J.A.M. (2019). Outcome prediction in postanoxic coma with deep learning. Crit. Care Med..

[b0225] Traub S.J., Wijdicks E.F. (2016). Initial diagnosis and management of coma. Emerg. Med. Clin. North Am..

[b0230] Tzovara A., Rossetti A.O., Juan E., Suys T., Viceic D., Rusca M., Oddo M., Lucia M.D. (2016). Prediction of awakening from hypothermic postanoxic coma based on auditory discrimination: Awakening from Postanoxic Coma. Ann. Neurol..

[b0235] van Leeuwen K.G., Sun H., Tabaeizadeh M., Struck A.F., Van Putten M.J.A.M., Westover M.B. (2019). Detecting abnormal electroencephalograms using deep convolutional networks. Clin. Neurophysiol. Off. J. Int. Fed. Clin. Neurophysiol..

[b0240] Vassallo P., Novy J., Zubler F., Schindler K., Alvarez V., Rüegg S., Rossetti A.O. (2021). EEG spindles integrity in critical care adults. Analysis of a randomized trial. Acta Neurol. Scand..

[b0245] Westhall E., Rossetti A.O., van Rootselaar A.-F., Wesenberg Kjaer T., Horn J., Ullén S., Friberg H., Nielsen N., Rosén I., Åneman A., Erlinge D., Gasche Y., Hassager C., Hovdenes J., Kjaergaard J., Kuiper M., Pellis T., Stammet P., Wanscher M., Wetterslev J., Wise M.P. (2016). Standardized EEG interpretation accurately predicts prognosis after cardiac arrest. Neurology.

[b0250] You W., Tang Q., Wu X., Feng J., Mao Q., Gao G., Jiang J. (2018). Amplitude-integrated electroencephalography predicts outcome in patients with coma after acute brain injury. Neurosci. Bull..

[b0255] Young G.B. (2000). The EEG in coma. J. Clin. Neurophysiol. Off. Publ. Am. Electroencephalogr. Soc..

[b0260] Zafar S.F., Rosenthal E.S., Jing J., Ge W., Tabaeizadeh M., Aboul Nour H., Shoukat M., Sun H., Javed F., Kassa S., Edhi M., Bordbar E., Gallagher J., Moura V., Ghanta M., Shao Y., An S., Sun J., Cole A.J., Westover M.B. (2021). Automated annotation of epileptiform burden and its association with outcomes. Ann. Neurol..

[b0265] Zhang Y., Su Y.Y., Haupt W.F., Zhao J.W., Xiao S.Y., Li H.L., Pang Y., Yang Q.L. (2011). Application of electrophysiologic techniques in poor outcome prediction among patients with severe focal and diffuse ischemic brain injury. J. Clin. Neurophysiol. Off. Publ. Am. Electroencephalogr. Soc..

[b0270] Zubler F., Koenig C., Steimer A., Jakob S.M., Schindler K.A., Gast H. (2016). Prognostic and diagnostic value of EEG signal coupling measures in coma. Clin. Neurophysiol..

[b0275] Zubler F., Steimer A., Kurmann R., Bandarabadi M., Novy J., Gast H., Oddo M., Schindler K., Rossetti A.O. (2017). EEG synchronization measures are early outcome predictors in comatose patients after cardiac arrest. Clin. Neurophysiol..

