# Supplementary material for: Diagnostic and prognostic EEG analysis of critically ill patients: A deep learning study
Source: Neuroimage Clin. 2022 Aug 27;36:103167. doi: 10.1016/j.nicl.2022.103167 (PMC9441331; doi:10.1016/j.nicl.2022.103167)
Supplement: Supplementary Data 1 [file mmc1.docx]

Supplementary Material

**1. Architecture and training**

*1.1 Detailed network architecture*

The t-VGG GAP network used in the present study consisted of three blocks (each containing two convolutional and one max-pooling layer), one Global Averaging Pooling layer, followed by one output layer. The output layer consisted of a single neuron (with sigmoid activation function) when predicting the outcome; it consisted of four neurons (with softmax activation function) when predicting the etiology.

**Table S1: Blocks and GAP**

|  | Layer | Description |
| --- | --- | --- |
| Block 1 | Input | 1x500x9, corresponding to 10 seconds of EEG at 50 Hz, with nine channels |
|  | 1-D Convolution | 16 filters, kernel length 3, stride 1 |
|  | BatchNormalization |  |
|  | Activation | ReLU |
|  | 1-D Convolution | 16 filters, kernel length 3, stride 1 |
|  | BatchNormalization |  |
|  | Activation | ReLU |
|  | Max-Pooling | pool size 4, stride 4 |
|  |  |  |
| Block 2 | 1-D Convolution | 32 filters, kernel length 3, stride 1 |
|  | BatchNormalization |  |
|  | Activation | ReLU |
|  | 1-D Convolution | 32 filters, kernel length 3, stride 1 |
|  | BatchNormalization |  |
|  | Activation | ReLU |
|  | Max-Pooling | pool size 4, stride 4 |
|  |  |  |
| Block 3 | 1-D Convolution | 32 filters, kernel length 3, stride 1 |
|  | BatchNormalization |  |
|  | Activation | ReLU |
|  | 1-D Convolution | 32 filters, kernel length 3, stride 1 |
|  | BatchNormalization |  |
|  | Activation | ReLU activation |
|  | Max-Pooling | pool size 4, stride 4 |
|  |  |  |
| GAP | Global Average Pooling |  |
|  |  |  |
| Output Layer  Setting:  Prognostication | Fully-connected Layer | 1 neuron, sigmoid activation |
|  | OR |  |
| Setting:  Etiology | Fully-connected Layer | 4 neurons, softmax activation |

*1.2. Training procedure*

We performed a stratified 5-fold cross validation, each time using four folds for training and the remaining fold as test set. The stratified split ensures that each fold contains roughly the same class distribution. In the binary setting (outcome/survival) the loss function to be minimized by the optimizer was the binary cross-entropy; in the multi-class setting (etiology), the loss function was the sparse categorical cross-entropy. We optimized our model using the Adam optimizer with a learning rate of 2e-5 (binary) or 1e-4 (multi-class). To counter-act the class imbalances, we also calculated the class weights of the training set, which were given as parameter to the Keras model fitting call. This parameter setting tells Keras to weigh the loss function relative to the class occurrences. The number of trainable parameters was 12’305 in the binary setting and 12’404 in the etiology setting. See source code below.

**2. Source Code**

*2.1 t-VGG GAP Model Implementation*

'''

This method returns the compiled "tVGG GAP" model used in the binary class setting (prognostication).

It is a 1D-convolutional network for EEG epoch inputs of the shape (timeseries_windowsize, number of channels)

The model architecture is adapted from the paper “EEG-based Outcome Prediction after Cardiac Arrest with Convolutional Neural Networks:

Performance and Visualization of Discriminative Features” by Jonas et al. Human Brain Mapping 2019 where further information can be found.

Keras v2.3.1 with a TensorFlow backend

'''

def compile_single_class_TVGG_model(input_vars=(500,9), learningrate = 0.0001):

    timeseries_windowsize = input_vars[0]

    num_channels = input_vars[1]

    model = Sequential()

    # Block 1

    model.add(Conv1D(16, kernel_size=3,strides=1,

                     name="firstCV",use_bias=False,

                     kernel_initializer="glorot_uniform", input_shape=(timeseries_windowsize,num_channels)))

    model.add(BatchNormalization())

    model.add(Activation('relu'))

    model.add(Conv1D(16, kernel_size=3,strides=1, use_bias=False, kernel_initializer="glorot_uniform"))

    model.add(BatchNormalization())

    model.add(Activation('relu'))

    model.add(MaxPooling1D(pool_size = 4, strides=4))

    # Block 2

    model.add(Conv1D(32, kernel_size=3,kernel_initializer="glorot_uniform",

                     strides=1, use_bias = False))

    model.add(BatchNormalization())

    model.add(Activation('relu'))

    model.add(Conv1D(32, kernel_size=3,strides=1, use_bias = False))

    model.add(BatchNormalization())

    model.add(Activation('relu'))

    model.add(MaxPooling1D(pool_size = 4, strides=4))

    # Block 3

    model.add(Conv1D(32, kernel_size=3,kernel_initializer="glorot_uniform",strides=1, use_bias = False))

    model.add(BatchNormalization())

    model.add(Activation('relu'))

    model.add(Conv1D(32, kernel_size=3,strides=1, use_bias=False, name="lastCV")) # the name is used as referral for GradCAM

    model.add(BatchNormalization())

    model.add(Activation('relu'))

    model.add(MaxPooling1D(pool_size = 4, strides=4))

    # Global Average Pooling operation after the third block

    model.add(GlobalAveragePooling1D())

    # into a final single-neuron FC layer outputting the probability

    model.add(Dense(1, activation='sigmoid'))

    adam = optimizers.Adam(lr=learningrate)

    model.compile(loss='binary_crossentropy',

              optimizer=adam,

                metrics=['accuracy'])

    return model

'''

Returns the same model architecture compiled and adjusted for the multi-class (etiology, 4 classes) setting.

'''

def compile_multi_class_TVGG_model(input_vars=(500,9), learningrate = 0.0001, num_final_classes = 4):

    timeseries_windowsize = input_vars[0]

    num_channels = input_vars[1]

    model = Sequential()

    # Block 1

    model.add(Conv1D(16, kernel_size=3,strides=1,

                     name="firstCV",use_bias=False,

                     kernel_initializer="glorot_uniform", input_shape=(timeseries_windowsize,num_channels)))

    model.add(BatchNormalization())

    model.add(Activation('relu'))

    model.add(Conv1D(16, kernel_size=3,strides=1, use_bias=False, kernel_initializer="glorot_uniform"))

    model.add(BatchNormalization())

    model.add(Activation('relu'))

    model.add(MaxPooling1D(pool_size = 4, strides=4))

    # Block 2

    model.add(Conv1D(32, kernel_size=3,kernel_initializer="glorot_uniform",

                     strides=1, use_bias = False))

    model.add(BatchNormalization())

    model.add(Activation('relu'))

    model.add(Conv1D(32, kernel_size=3,strides=1, use_bias = False))

    model.add(BatchNormalization())

    model.add(Activation('relu'))

    model.add(MaxPooling1D(pool_size = 4, strides=4))

    # Block 3

    model.add(Conv1D(32, kernel_size=3,kernel_initializer="glorot_uniform",strides=1, use_bias = False))

    model.add(BatchNormalization())

    model.add(Activation('relu'))

    model.add(Conv1D(32, kernel_size=3,strides=1, use_bias=False, name="lastCV"))

    model.add(BatchNormalization())

    model.add(Activation('relu'))

    model.add(MaxPooling1D(pool_size = 4, strides=4))

    # GAP into a single output layer

    model.add(GlobalAveragePooling1D())

    # num_classes neurons activated by softmax

    model.add(Dense(num_final_classes, activation='softmax'))

    adam = optimizers.Adam(lr=learningrate)

    model.compile(loss='sparse_categorical_crossentropy',

              optimizer=adam,

                metrics=['accuracy'])

    return model

*2.2 GradCAM Implementation*

'''

The GradCAM method implementations.

Python v3.7.1

Keras v2.3.1 with a TensorFlow backend

'''

from keras import backend as K

from tensorflow.keras import Model

'''

This method returns the GradCAM values for a selected class in the binary setting.

Inputs:

    model = The fully trained and saved model for classification

    EEG_epoch = The specific 1D epoch to obtain the values for

    timeseries_windowsize = length in datapoints of the epoch

    numChannels = # of channels in the input epoch

    selected_class = Obtain values for class 0 or class 1 (binary)

    layer_name = The layer name in the model (pre-defined) from which the gradients will be obtained (usually the last)

Returns:

    gc_values = An array with the gradcam values which can then be further resized and edited for visualizations.

In our case the EEG epoch is of shape (500, 9), the last convolutional layer is named "lastCV" and the selected class is 1.

'''

def get_gradcam_singleclass(model, EEG_epoch, timeseries_windowsize, numChannels, selected_class=1, layer_name="lastCV"):

    y_c = model.output[0]

    conv_layer = model.get_layer(layer_name)    # defined in the saved model

    gradcam_model = Model([model.inputs], [conv_layer.output, model.output])

    # Get gradient w.r.t. the output of the selected conv layer

    with tf.GradientTape() as gtape:

        conv_output, predictions = gradcam_model(EEG_epoch.reshape(1, timeseries_windowsize, numChannels))

        loss = predictions[0]

        grads = gtape.gradient(loss, conv_output)

        pooled_grads = K.mean(grads, axis=(0, 1))

    if selected_class == 0:

        pooled_grads = pooled_grads * -1

    gc_values = tf.reduce_mean(tf.multiply(pooled_grads, conv_output), axis=-1)

    gc_values = gc_values[0]

    gc_values = np.maximum(gc_values,0)

    gc_values /= np.max(gc_values) # optional norm.

    return gc_values

'''

This method was used to retrieve the gradCAM values in the multi-class setting, i.e. the etiology setting.

Changed inputs:

    selected_class = Possibilities of 0,1,2 or 3 in the 4-class setting.

'''

def get_gradcam_multiclass(model, EEG_epoch, timeseries_windowsize, numChannels, selected_class=2, layer_name = "lastCV"):

    conv_layer = model.get_layer(layer_name)

    gradcam_model = Model([model.inputs], [conv_layer.output, model.output])

    with tf.GradientTape() as gtape:

        conv_output, predictions = gradcam_model(EEG_epoch.reshape(1, timeseries_windowsize, numChannels))

        loss = predictions[:,selected_class]

        grads = gtape.gradient(loss, conv_output)

        pooled_grads = K.mean(grads, axis=(0, 1))

    gc_values = tf.reduce_mean(tf.multiply(pooled_grads, conv_output), axis=-1)

    gc_values = gc_values[0]

    gc_values = np.maximum(gc_values,0)

    gc_values /= np.max(gc_values) # optional norm.

    return gc_values

*2.3 Training Procedure*

import numpy as np

from sklearn.utils import class_weight

from sklearn.model_selection import StratifiedKFold

'''

This snippet showcases the training procedure applied in our study.

Dataset-specific processing steps are omitted.

Python v3.7.1 and Keras v2.3.1 with a TensorFlow backend

sklearn v0.20.3 ; numpy v1.18.5

'''

# ... pre-processing, data-splitting and loading:

EEG_DATALIST = # a list of patient-eeg names

EEG_LABELLIST = # a list containing the corresponding label to each eeg

# 5-fold stratified cross-Validation:

# (omitted: prepare documentation for each fold)

skf = StratifiedKFold(n_splits=5, random_state=CV_RANDOM_STATE, shuffle=True)

for train_index, test_index in skf.split(EEG_DATALIST, EEG_LABELLIST):

  # create training sets (4 folds) and a test set (1 CV fold)

  train_eeg_list, test_eeg_list = EEG_DATALIST[train_index], EEG_DATALIST[test_index]

  x_train, y_train, ids_train = # create_dataset(train_eeg_list, ...) - load the epochs

  x_test, y_test, ids_test = # create_dataset(test_eeg_list, ...)

  # load a compiled "t-VGG GAP" model. Here: single-class model, see further supplied material.

  lr = 0.00002

  model = eeg_models.compile_single_class_TVGG_model(input_vars=(500,9), learningrate = lr)

  # sidestep: counter-act class imbalances, supply class weights to training call (see model.fit)

  class_weights = class_weight.compute_class_weight('balanced',np.unique(y_train),y_train)

  class_weight_dict = {}

  class_weight_dict[0], class_weight_dict[1] = class_weights[0], class_weights[1]

  # fit and train the model

  # the model was pre-compiled with the parameters:

  # Optimizer: Adam; loss function: binary cross-entropy

  callback = modelhelper.get_earlytraining_callback(training_stop = ts)

  model.fit(x_train, y_train, batch_size=bs,

              epochs=max_epochs, verbose=1,shuffle=True,

                callbacks=callback, class_weight=class_weight_dict)

  # omitted: documentation and saving the model (a saved state is needed for Grad-CAM)

  # model.save(model_name)

  # model evaluation

  acc, sensitivity, specificity, PPV, NPV, roc_auc = # evaluate_model(test_eeg_list, model=model, ...)

  # omitted: further documentation

  # reset the loaded and compiled model:

  K.clear_session()

  model = None
